# The Data Age Is on the Verge of Full Bloom

**Published:** 2011

**Authors:** Soroush Sardari

As the medical sciences in part of human civilization is developing and humans distancing themselves from other sorts of life, rightfully or not, existence of certain tangible or metaphysical items can impact human definition. One of such influential items is “data”. By data creation, storage, manipulation and transfer, we can certainly define ourselves, as “We have data, so we exist”. Apart from the fact that too much focus on any dogma in creation, which could lead to a tunnel-vision mentality; ignoring the importance and significance of this growth and influential bio-medical scientific scaffold, would result in shear ignorance and failure. Therefore, scientific community is likely one of the first sectors whom are exposed to and feel the sheer force of the “tsunami-like” waves of “data-age”.

Data era has signs and symptoms that prove it has appeared. The numbers, at large, in the scientific community, are indicative of a huge volume of generated knowledge and establishment of departments or research units in pharmaceutical and medical schools as bioinformatics or its relatives came in response to a head-to-head need in absorbing the “data-shock”. While it used to be sufficient for a project to start with a background check in only one database of hardly existing few choices, in 10-20 years ago; it is by no means, the case now. The present era is coloring itself in the “flood of data”. A student in any graduate program can never be sure of the final and comprehensive status of the research in a particular field. Let alone, the professor, whom has delivered the subject of the project or the title, is in the similar situation. Starting a literature search to begin a project in any academic setting, has to be simultaneously carried out in at least three databases, in addition to a handful of search engines. Yet, it may be a common scene to see many surprised students and professors to encounter an existing paper or recently published results, of competing nature, right after spending the entire project budget and preparing to compile the draft of their paper. All these observations prove the critical situation for “data flood” phenomenon and underline the absolute need for awareness and wisely prepared steps to be taken in order to manage data, find the proper tools to handle it and prevent possible losses, particularly in the growing fields of biomedical and pharmaceutical sciences. It is evident that preparedness does not get along with traits of certain nature like hiding the truth, denial, self-proclaim, institute-centered mentality, etc. It rather flourishes after honest fact-finding and *status quo *reports, followed by careful and practical planning. Only after such meaningful stages are covered, comes the turn for developing new tools in the data management or rightfully borrowing the ones invented and used earlier by other institutions or organizations, rather than losing the time by re-inventing the wheel. 

Of course, just because there is more information, it does not mean that we are consuming it. First and foremost, data must be available, and then we need to have tools to process and digest it. Data access in the current of changing world is as essential as water in a desert; providing access is just the beginning to forming a civilization in the global-desert. This is based on the central water-like role of data, on the survival of any form of life in a world that relies more and more on data and thrives to produce information, knowledge and progress. Access to data will empower investment. It is not enough to rely on older forms with limited availability such as regular internet data; the specialized groups of each civil society require their own requirement to reach the desired forms of access in a manageable, statistically analyzable shape. Only after enough access to water, comes the concept of hygiene; data-cleansing is part of data management. It may seem counter intuitive, when we consider that data-cleansing an indispensible form of data management. An analogy to better understand this homology refers to identifying and removing the junk data from any statistical study or accumulating samples or repeating measurements to enhance valid data points, while reducing the noises.

Access to data will lead to creation of new data and productions of information that is the high ranking version of data. This is data cultivation, as if watering various types of trees would produce juicy fruits of multiple colors and properties. Creation of more data, enhances the usage and diversity in tastes, which leads to evolving the better strains of data and eliminating the poor quality ones. This cycle which is in accordance to natural phenomena, assures the well-being of knowledge and strengthens the science foundation at its best. All of these, together with delicate care of “data-garden” are the results of availability and existence of various pointed attributes for a fruitful and usable data-utility to appear from science to application. 

*Soroush Sardari* is currently working as an assistant professor of Pharmacognosy at *Medical Biotechnology Department, Biotechnology Research Center, Pasteur Institute, Tehran 13164, Iran* could be reached at the following e-mail address*:* *ssardari@hotmail.com*

